# Supplementary material for: Identification of photoperiod-induced specific miRNAs in the adrenal glands of Sunite sheep (Ovis aries)
Source: Front Vet Sci. 2022 Jul 22;9:888207. doi: 10.3389/fvets.2022.888207 (PMC9354845; doi:10.3389/fvets.2022.888207)
Supplement: Supplementary file 1 [file Data_Sheet_1.ZIP › Supplementary file/miRDeep2 file.zip!/miRDeep2 file/Know_Structure/SPLP42A2/oar-mir-668.pdf]

miRBase precursor : oar-mir-668
Total read count : 45
oar-miR-668-5p read count : 1
oar-miR-668-3p read count : 44
remaining reads : 0

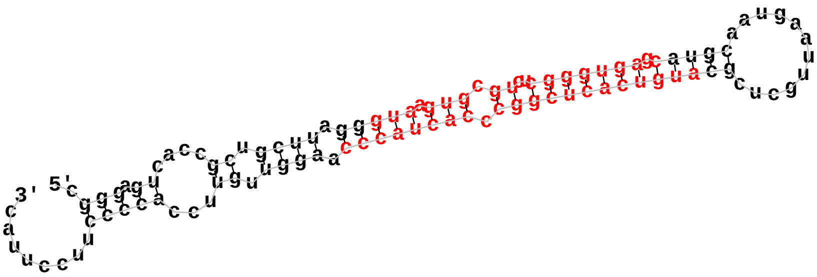

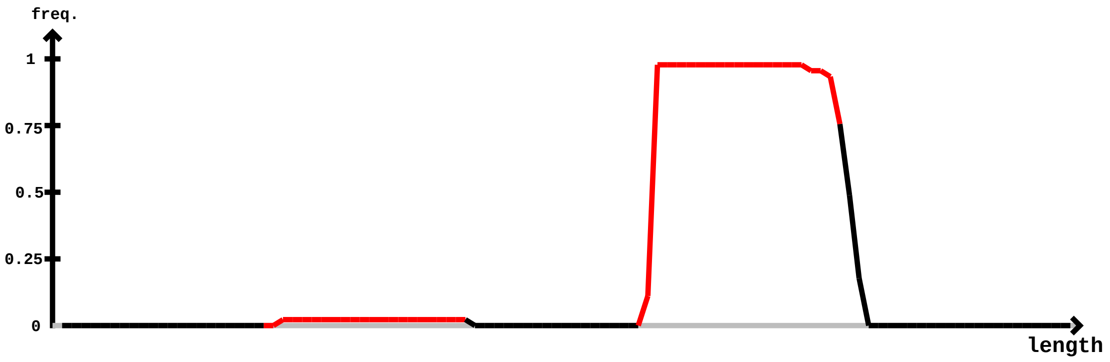


```
                                                           oar-miR-668-3p
                     oar-miR-668-5p
5'- cgggagucaccgcugcuuaggguaagugcgucucgggugagcaugcaaugaauugcucgcaugucacucggcccacuacccaagguuguuccaccccuuccuuac   -3'    exp
    .(((.((....((.((((.(((((.(((.((..(((((((.(((((............))))))))))))))).)))))))).)))).))...)))))........    reads      mm        sample
    ......................uaagugcgucucgggugagc...............................................................    1          0         seq
    ............................................................augucacucggcccacu............................    1          0         seq
    ............................................................augucacucggcccacuaccc........................    2          0         seq
    ............................................................augucacucggcccacuaccca.......................    2          0         seq
    .............................................................ugucacucggcccacuac..........................    1          0         seq
    .............................................................ugucacucggcccacuacc.........................    8          0         seq
    .............................................................ugucacucggcccacuaccc........................    10         0         seq
    .............................................................ugucacucggcccacuaccca.......................    12         0         seq
    .............................................................ugucacucggcccacuacccaa......................    8          0         seq
```
